# COPD at the end of life: Predictors of the emotional distress of patients and their family caregivers

**DOI:** 10.1371/journal.pone.0240821

**Published:** 2020-10-16

**Authors:** Ana Soto-Rubio, Selene Valero-Moreno, José Luis Díaz, Yolanda Andreu, Marián Pérez-Marín

**Affiliations:** 1 Personality, Assessment, and Psychological Treatments Department, Faculty of Psychology, University of Valencia, Valencia, Spain; 2 Air Liquide Healthcare, Valencia, Spain; 3 Respiratory Care Unit, Hospital Clínico Universitario de Valencia, Valencia, Spain; 4 International University of Valencia (VIU), Valencia, Spain; University of Milano Biococca, ITALY

## Abstract

**Background:**

Few studies have focused on patients' emotional distress with end-stage chronic obstructive pulmonary disease (COPD) and their main family caregivers.

**Methods:**

Cross-sectional data about emotional, functional, and burden-related variables were collected from 85 patients with end-stage COPD and their 85 respective main family caregivers to determine the variables that could predict their emotional well-being. Descriptive analyses, comparison of means, hierarchical regression models, and comparative quali-quantitative analyses were carried out.

**Results:**

Data show that the great majority of patients with COPD spend years with this diagnosis, and have been admitted to the hospital several times in advance stage of illness the previous year of the moment of end-of-life stage. Furthermore, only a tiny percentage of the patients were functionally independent in the advanced stage of illness.

**Conclusions:**

The emotional distress and the burden of the family caregiver play an essential role in the distress of the patient, in conjunction with the patient's own functional independence and the time living with the disease, and comorbidity. On the other hand, variables of the patient, such as time since diagnosis, number of hospital admissions, comorbidity, functional dependence, and emotional distress, play an important role in the family caregiver's emotional distress and burden. Understanding how these variables are related is key to designing appropriate programs to reduce the emotional distress the patients with COPD at the end of life and their family caregivers.

## Introduction

End-of-life care poses a series of medical, emotional, social, and spiritual challenges [1] to the patient and their family [2]. Family members may be continually worried about the patient’s health [3]. The reality of providing care may also place a heavy burden on the family [2, 4].

When faced with a family member's severe illness, a "main caregiver” emerges in most families: “people who regularly provide the most assistance with one or more of the core activities of communication, mobility, transport, housework, and self-care” [5]. The main family caregivers' dedication also tends to go beyond the actual time spent providing care, being permanently “on-call" for any patient's needs. The consequent loss of control over their time is a significant factor in the caregivers’ perception of their burden [4, 6].

Several studies examining family caregivers at the end of life identified that they provide more than half of the patient's care in the late stage of life [5, 7–9]. In most cases, these carers are women, typically the patient’s daughter or wife [4, 10, 11].

Taking on this role of the main family caregiver may have adverse effects on the patient's and family members' health [12–14]. The majority of family caregivers also need care themselves and can be thought of as “hidden patients” [15].

Symptoms of anxiety and depressions are frequently reported by family caregivers of highly dependent patients [14, 16, 17]. The perceived burden has an enormous impact on the degree of depression the family caregiver may suffer [18, 19]. Younger family members tend to experience more significant emotional distress, whereas older people find it harder to undertake the tasks that taking care of their family member involves [10, 11]. The disease's specific characteristics may also pose a series of challenges for caregivers and patients [20]. The time during which the caregiver provides care and the severity of the symptoms may impact the degree of depression they may suffer [10, 11]. However, some studies indicate that it may not be the disease's duration overall, but more specifically, the terminal phase duration, associated with a significant emotional impact on family caregivers [21].

The patient’s emotional state is another factor that research has shown to be closely linked to the family caregiver’s emotional state [22, 23].

Concerning end-stage chronic obstructive pulmonary disease (COPD), some studies address the relationship between the disease's psychological and physical aspects [24–26]. However, very few studies focus on these patients' emotional distress in the end-of-life stage and their main family caregivers as a dyad. The available data are found in studies of family caregivers of patients with a wide range of pathologies, including COPD [5, 27]. It is important to note that COPD is characterized by long, progressive deterioration interrupted by periodic crises, which alternate with periods of stability. Death finally comes in a relatively sudden manner [28]. This alternation between periods of acute crises and periods of stability (as well as the unpredictability of the death) makes the emotional toll on patients and their families particularly intense since the disease's trajectory influences the emotional adaptation of the family caregiver [29]. Some studies have indicated that, in the case of patients suffering severe breathlessness, their family caregivers report fewer positive, caring experiences than family caregivers of other types of patients [30]. Also, the family caregiver's depression level is particularly affected by how long the caregiving tasks have been performed and the severity of the d [31, 32]. Finally, numerous studies have shown how family caregivers of patients with a long duration progressive illness such as COPD present a greater risk of problems concerning their mental or physical health, emotional distress, and an increase in mortality [4, 33].

For all these reasons, it is essential to pay due attention to the predictors of emotional distress in family caregivers and patients at the end of life, to reduce their suffering and prevent future pathologies.

### Objectives

To study the relationship between the variables linked to the emotional distress in end-stage COPD patients and their main family caregivers.

## Material and methods

### Participants

Between 2016 and 2018, cross-sectional data on 85 patients with end-stage COPD and their 85 respective main family caregivers were collated, the participants were not compensated. All caregivers were considered primary and had a family connection to the patient. 95.3% of the patients were male (age range = 43–95, *M* = 73.46; *SD* = 9.59), 9.6% of the family caregivers were male (age range = 22–83; *M* = 61.31; *SD* = 14.07). Regarding the patient, 50.6% had primary education, 38.8 5% no education, 7% high school, and 3.6% higher education. The kinship of the main family caregivers with the patient was as follows: 61.2% were partners or spouses, 29.4% were children, 2.4% were siblings, and 7.1% had other kinds of kinship (brothers-in-law, uncles). According to their education level, the main family caregivers' distribution was: 53.6% primary education, 22.6% without education, 16.6% high school, and 7.2% higher education. Regarding the employment situation, 49.4% of the family caregivers were retired, 21.2% were working, 16.5% were unemployed, and 12.9% were in other situations (self-employed).

### Variables and instruments

- The main sociodemographic characteristics of the patient and their main family caregiver, as well as time since diagnosis, number of hospital admissions during the previous year, and comorbidity were recorded using an ad hoc interview.
- The patient’s functional independence was assessed using the Barthel Index [34]. This scale was initially designed to assess and monitor progress in patients' self-care independence with neuromuscular or musculoskeletal pathology, and it has shown its usefulness for patient assessment in general. In Spain, the version [35] has contributed substantially to its dissemination and use. It includes assessing ten basic daily living activities such as eating, washing, dressing, grooming, bowel movements, urination, using the toilet, moving, wandering, and using steps. Scores range from 0 for maximum dependence to 100 for maximum independence.
- The emotional state of the patient and his or her family caregiver was assessed using the Hospital Anxiety and Depression Scale (HADS) [36] as adapted for the Spanish population [37]. This instrument is widely used in the adult clinical field to assess anxiety and depression symptomatology in people with physical diseases [38]. For each of the 14 items, the patient must select the answer that best reflects their situation during the previous week. Each subscale score is obtained by adding up the selected statements (0–3) on the items (even items for depression, odd items for anxiety). For example: "I have lost interest in my personal appearance" for depression, or “I feel restless, like I cannot stop move" for anxiety. The range of scores is 0–21 for each subscale, and 0–42 for the overall score. Previous studies have shown good psychometric properties for this scale [36, 39, 40]. In the present work, adequate indices were also obtained for the sample of main family caregivers (anxiety *α* = .88, depression *α* = .85 and emotional distress α = .90) and the sample of patients (anxiety *α* = .81, depression *α* = .80 and emotional distress *α* = .83).
- The burden of the main family caregiver was assessed using the Zarit Burden Interview [41]. It is a self-report questionnaire designed to measure the level of caregiver burden. This questionnaire contains 22 items ("Do you feel that your family member is asking for more help than he or she really needs?"), each of which is a question that the caregiver must answer using a 5-point Likert scale, where 1 = never, and 5 = almost always. The total score is the sum of all items, and it ranges from 22 to 110. Previous studies have shown good psychometric properties [42, 43]. In this study, for the sample of family caregivers *α* = .87.

### Procedure

All patients had been admitted to a respiratory care unit at the time of assessment. The palliative care team of each hospital informed those patients considered by the medical staff in the end-of-life stage and their families about the study. The inclusion criteria for patients were:

- To have COPD as the principal diagnosis.
- To be considered in the end-of-life stage by the medical team, following the criteria established by the Spanish Society of Palliative Care [44]:
  ○ Presence of an advanced, progressive, incurable disease.
  ○ Lack of reasonable possibilities of response to the specific treatment.
  ○ Presence of numerous intense, multiple, multifactorial, and changing physical problems or symptoms.
  ○ Limited life expectancy.

The inclusion criterion for family caregivers was:

- To be identified by a patient participating in the study as their main family caregiver. The following definition was used to describe the main family caregiver: “the member of the family who regularly provides the most assistance with one or more of the core activities of communication, mobility, transport, housework, and self-care."

The exclusion criterion for patients and family caregivers was to present significant cognitive impairment, as assessed with the Short Portable Mental Status Questionnaire [45].

When a medical team member identified a patient and a family caregiver who met the inclusion criteria, they were considered a potential participant. The medical team determined which patients were at the end of life and which did not show cognitive impairment. A psychologist collated the rest of the data via a face-to-face interview, which took place during the patient's first week of admission at the *Hospital Clínico Universitario de Valencia*'s respiratory care unit. Each participant signed an informed consent form and a confidentiality agreement following the Declaration of Helsinki principles. The assessment protocol was approved by the ethics committee of the Universitat de València (H1385291905651).

### Data analysis

First, the descriptive analyses, the comparison of means, and the analysis of relations were carried out. The hierarchical regression models (HRM) and the quali-quantitative comparative analysis (QCA) were then performed.

The QCA allows the quantitative analysis of a small number of cases, using Boolean algebra as a formal tool to identify which of a series of factors (independent variables or causal conditions) are associated with the presence of a given result (criterion variable or result condition). Thus, it allows proposing pathways (which combine a particular interaction between the variables) to optimize the prediction of the independent variable [46]. The QCA analysis establishes the so-called "necessary causes," which must always be present for a result to be given; and the "sufficient conditions," which do not always have to be present to produce a result but sill they can combine to generate a specific result. QCA models allow the identification of the percentage of variance explained (the coverage) and the indicators of goodness of fit (the consistency) [47, 48]. Finally, the QCA analysis generates three possible solutions: complex, parsimonious, and intermediate. The literature recommends the use of the intermediate one [48], so this study provides it.

SPSS 24 was used to perform descriptive analyses, comparison of means, Pearson's correlations, and the hierarchical regression model, while fsQCA 3.0 was used to perform the QCA analyses.

## Results

### Descriptive analysis

All patients had COPD. The time since diagnosis in years varied from 1 to 40 years, although approximately the average time was 9 years (*M* = 8.92; *SD* = 7.20).

The number of admissions in the last year ranged from 1 to 21 (*M* = 6.30; *SD* = 5.24). The duration of these admissions ranged from 1 to 30 days (*M* = 5.31; *SD* = 4,61).

On the other hand, although all patients presented COPD, most showed comorbidity with other diagnoses, approximately patients showed three additional diagnoses of COPD. The range of comorbidity varied between 0 and 7 (*M* = 2.91; *SD* = 1.67). The most frequent diagnoses in patients were: 42.4% hypertension; 28.2% type 2 diabetes mellitus, and 21.2% dyslipidemia. Also, 11.8% smoked. Regarding the level of functional independence, measured by the Barthel index, 10.7% presented functional independence, 67.9% slight dependency, 8.3% moderate dependency, 8,3% severe dependency, and 4.8% total dependence.

### Comparison of means and relations

The comparison of means, and relation analysis according to kinship were performed, but no statistically significant outcome was found. This same type of analysis of the data could not be carried out taking into consideration the gender of the participants since the variability was not big enough (the great majority of family caregivers were women and almost all the patients were men).

Finally, the patient and family caregiver psychological variables were related to the medical variables of the patient: the patient's emotional distress was negatively related to functional independence (*r* = -.31; *p*≤.01) and positively related to the family caregiver distress (*r* = .25; *p* = .02) and family caregiver burden (*r* = .24; *p* = .03). The family caregiver's emotional distress was positively related to his/her burden (*r* = .38; *p*≤ .001).

### Hierarchical regression models (HRM)

Two hierarchical regression models were tested to analyze the predictive power of the variables under study. The criterion variable in model 1 was the emotional distress of the patient and, in model 2, it was the emotional distress of the family caregiver. The predictor variables considered in both models were: medical variables of the patient (time since diagnosis, number of hospital admissions, comorbidity, and functional independence level), psychological variables of patients (emotional distress), and psychological variables of the family caregivers (emotional distress and burden).

In model 1 (prediction of emotional distress of the patient), two differential steps were established: first, the family caregiver variables (emotional distress and burden), and second, the medical variables of the patient (time since diagnosis, number of hospital admissions, comorbidity and functional independence level). In the first step of this first model (prediction of the patient's emotional distress), the family caregiver variables increased the explained variance by 8% (ΔR^2^ = .08; *p* = .03). In the second step, the patient's medical variables increased the explained variance by 15% (ΔR^2^ = .15; *p* = .01). In this last step, the significant variables were: functional independence level (*β* = -.23; *p*≤ .05) and time since diagnosis (*β* = .26; *p* = .03), explaining in total a 17% of the variance of the emotional distress of patient (Table 1).

**Table 1:** Hierarchical regression model.

**Predictors**	**Emotional distress of patient**			
	*ΔR*^*2*^ ^*b*^	*ΔF* ^*c*^	ß ^d^	*t* ^*e*^
Step 1	.08*	3.55		
Emotional distress of caregiver			.13	1.20
Burden of caregiver			.13	1.12
Step 2	.15**	3.51		
Time since diagnosis			.26*	2.29
Number of hospital admissions			-.19	-1.71
Comorbidity			.14	1.34
Functional Independence level			-.23*	-2.04
*Durbin-Watson*	2.11			
*R*^*2*^_*ajd*_^*a*^	.17**			
	**Emotional distress of family caregiver**			
	*ΔR*^*2*^ ^*b*^	*ΔF* ^*c*^	ß ^d^	*t* ^*e*^
Step 1	.03	.53		
Time since diagnosis			.11	0.93
Number of hospital admissions			-.09	-0.76
Comorbidity			-.01	-0.11
Functional Independence level			.06	0.53
Step 2	.04	3.09		
Emotional distress of patient			.14	1.20
Step 3	.12	10.72**		
Burden of caregiver			.36**	3.27
*Durbin-Watson*	1.96			
*R*^*2*^_*ajd*_^*a*^	.12*			

^a^ r adjusted square.

^b^ change in R2.

^c^ change in F.

^d^ regression coefficient.

^e^ value of t-test statistic.

_*p ≤ 0.05_

_**p ≤ 0.01_

_***p ≤ 0.001._

In model 2 (prediction of emotional distress of the family caregiver), three steps were established. First, the medical variables (time since diagnosis, number of hospital admissions, comorbidity, and functional independence level) were included; secondly, the patient's emotional distress; and finally, in the third step, the family caregiver's burden. In the first step, the medical variables did not significantly increase the explained variance (ΔR^2^ = .03; *p* = .71); in the second step, the patient's emotional distress not increased the explained variance (ΔR^2^ = .04; *p* = .08); and finally, in the last step, the explained variance increased by 12% (ΔR^2^ = .12; *p*≤.01). In this last step, the only significant variable was the family caregiver's burden (*β* = .36 *p*≤ .01), explaining a total of 12% of the variance of the family caregiver's emotional distress (Table 1).

### Quali-quantitative comparative analysis (QCA)

First, the main calibration values for the variables studied were calculated. These calibration values are presented in Table 2.

**Table 2:** Values of calibration for the quali-quantitative comparative analysis (QCA).

	Variables of the patient (Medical and psychological)					Variables of caregivers	
	Comorbidity	Hospital admissions	Time since diagnosis	Functional independence of the patient	Emotional distress of the patient	Emotional distress of the caregiver	Burden of the caregiver
*M*^*a*^	2.91	6.30	8.92	73.15	12657892.67	18725548.80	48.54
*SD*^*b*^	1.66	5.24	7.20	24.74	24550714.76	36316390.42	15.46
Min^c^	0	1	1	10	16384	16384	23
Max^d^	7	21	40	100	150994944	2011326592	92
P10^e^	1	1	1.40	30	154828.80	107827.20	31
P50^f^	3	5	7	77.50	2239488	3359232	47
P90^g^	5	15	18	100	43764940.80	570425334.40	69.60

^a^_mean._

^b^_statistical deviation._

^c^_minimum._

^d^_maximum._

^e^_percentile 10._

^f^_percentile 50._

^g^_percentile 90_.

_Note: Mean values are shown as calibrated for the QCA analysis, not as means of the direct scores._

Afterward, the necessity and sufficiency analyses were carried out. The established criterion variables ("result condition," according to the QCA terminology) were: on the one hand, the emotional distress of the patient; and on the other hand, the emotional distress of his/her family caregiver. For both criterion variables, the predictor variables (“causal conditions”) were established as well: patient variables, both medical (time of diagnosis, number of hospital admissions, comorbidity, and functional independence level), and psychological (emotional distress). From the family caregiver, the psychological variables considered were emotional distress and burden.

#### Necessity analysis

No necessary condition was observed for the occurrence or non-occurrence of the patient's emotional distress (Table 3). Similarly, no necessary condition was observed for the occurrence or non-occurrence of the emotional distress of the family caregiver (Table 4), since the consistency was in all the cases smaller than .90 [48].

**Table 3:** Necessity analysis of the emotional distress of the patient.

	Emotional distress of the patient		~^a^ Emotional distress of the patient	
	Cons^b^	Cov^c^	Cons	Cov
Longer time since diagnosis	.65	.61	.59	.62
Shorter time since diagnosis	.60	.58	.63	.66
Higher number of hospital admissions	.55	.57	.59	.67
Lower number of hospital admissions	.68	.60	.62	.60
Higher comorbidity	.60	.64	.50	.59
Lower comorbidity	.62	.53	.79	.66
Higher levels of emotional distress of the caregiver	.77	.75	.45	.49
Lower levels of emotional distress of the caregiver	.48	.44	.77	.79
Higher burden of the caregiver	.64	.65	.50	.55
Lower burden of the caregiver	.56	.50	.68	.68
Functional independence of the patient	.58	.50	.74	.70
Functional dependence of the patient	.65	.69	.47	.55

^a^_absence of result condition._

_b consistency._

^c^_coverage._

_Necessary condition: Consistency ≥ .90._

**Table 4:** Necessity analysis of the emotional distress of the family caregiver.

	Emotional distress of the caregiver		~ ^a^ Emotional distress of the caregiver	
	Cons^b^	Cov^c^	Cons	Cov
Longer time since diagnosis	.70	.68	.54	.56
Shorter time since diagnosis	.54	.53	.69	.71
Higher number of hospital admissions	.60	.64	.53	.60
Lower number of hospital admissions	.62	.56	.68	.64
Higher comorbidity	.61	.66	.50	.58
Lower comorbidity	.62	.54	.70	.66
Functional independence of the patient	.64	.56	.70	.66
Functional dependence of the patient	.61	.66	.53	.61
Higher levels of emotional distress of the patient	.75	.77	.44	.48
Lower levels of emotional distress of the patient	.49	.45	.79	.77
Higher burden of the caregiver	.63	.65	.52	.57
Lower burden of the caregiver	.58	.53	.68	.66

^a^_absence of result condition._

_b consistency._

^c^_coverage._

_Necessary condition: Consistency ≥ .90._

#### Sufficiency analysis

Parting from the premise that in QCA a model is informative when the consistency is around or above .74 [47], the adequacy analyses and the resulting models for each of the dimensions were as follows:

a) Prediction of the emotional distress of the patient.

In predicting high levels of emotional distress of the patient, five pathways explained 60% of the cases with high levels of emotional distress (Total consistency = .85; Total coverage = .60). Of these pathways, the two most relevant paths were: higher levels of emotional distress of the family caregiver, higher functional dependence, and higher comorbidity of the patient (Raw coverage = .41; Consistency = .88) (Table 5). Another combination was higher levels of emotional distress and burden of the family caregiver and higher comorbidity of the patient (Raw coverage = .39; Consistency = .88).

**Table 5:** Summary of the three main sufficient conditions for the intermediate solution for the emotional distress of the patient.

*Limit of frequency*:*1*	Emotional distress of the patient			~ ^a^Emotional distress of the patient		
	*Consistency’s cut-off*: .*89*			*Consistency’s cut-off*: .*90*		
	**1**	**2**	**3**	**1**	**2**	**3**
Longer time since diagnosis					○	○
Higher number of hospital admissions			○^c^	**●**		**●**
Higher comorbidity	**●**^b^	**●**				
Higher levels of emotional distress of the caregiver	**●**	**●**	**●**	○	○	○
Higher burden of the caregiver		**●**	**●**		○	○
Functional independence of the patient	○		○	**●**	**●**	
Raw coverage	.41	.39	.32	.39	.33	.24
Unique coverage	.02	.05	.04	.08	.11	.02
Consistency	.88	.88	.92	.93	.92	.95
**Total consistency**			**.85**			**.90**
**Total coverage**			**.60**			**.60**

^a^_absence of result condition._

^b^_presence of causal condition._

^c^_absence of causal condition._

_Expected vector for emotional distress of the patients 1.1.1.1.1.0; Expected vector for_ ~_emotional distress of the patients 0.0.0.0.0.1 [__49__]._

In reference to the prediction of low levels of emotional distress in the patient, five pathways explained 60% of the cases with low levels of emotional distress (Total consistency = .90; Total coverage = .60). Of these pathways, the two most relevant paths were: lower emotional distress of the caregiver, higher number of hospital admissions and higher functional dependence of patient (Raw coverage = .39; Consistency = .93) (Table 5), and another pathway was the combination of lower emotional distress and lower burden of the family caregiver, lower functional dependence of patient and lower time since diagnosis (Raw coverage = .33; Consistency = .92).

b) Prediction of emotional distress in the main family caregiver.

In the prediction of high levels of emotional distress in the main family caregiver, five paths explained the 68% of the cases with high levels of emotional distress (Total consistency = .81; Total coverage = .68) (Table 6). Of these pathways, the two most relevant paths were: higher levels of emotional distress of patients and higher time since diagnosis (Raw coverage = .57; Consistency = .89) (Table 6), and higher levels of burden of the caregiver, higher levels of emotional distress of patient and higher functional dependence of the patient (Raw coverage = .40; Consistency = .85).

**Table 6:** Summary of the three main sufficient conditions for the intermediate solution for the emotional distress of the caregiver.

*Limit of frequency*:*1*	Emotional distress of the caregiver			~ ^a^Emotional distress of the caregiver		
	*Consistency’s cut-off*: .*89*			*Consistency’s cut-off*: .*90*		
	**1**	**2**	**3**	**1**	**2**	**3**
Longer time since diagnosis	**●**^b^		**●**	○	○	○
Higher number of hospital admissions				**●**	**●**	**●**
Higher comorbidity			**●**	○	○	
Higher levels of emotional distress of the caregiver	**●**	**●**	**●**		○	○
Higher burden of the caregiver		**●**	**●**		○	○
Functional independence of the patient		○^c^	○	○	**●**	○
Raw coverage	.57	.40	.31	.22	.20	.20
Unique coverage	.20	.06	.01	.01	.06	.01
Consistency	.89	.85	.93	.90	.91	.92
**Total consistency**			**.81**			**.87**
**Total coverage**			**.68**			**.48**

^a^_absence of result condition._

^b^_presence of causal condition._

^c^_absence of causal condition._

_Expected vector for emotional distress of caregivers 1.1.1.1.1.0; Expected vector for_ ~_emotional distress of caregivers.0.0.0.0.1 [__49__]._

Finally, regarding the prediction of low levels of emotional distress in the main family caregiver, six pathways explained the 48% of the cases with low levels of emotional distress (Total consistency = .88; Total coverage = .48). Of these pathways, the two most relevant paths were: lower levels of emotional distress of patients, lower functional dependence, lower time since diagnosis, and lower comorbidity (Raw coverage = .25; Consistency = .89). Another combination was lower levels of caregivers' burden, lower levels of emotional distress of patients, lower functional dependence of patients, higher time since diagnosis and lower hospital admissions, and comorbidity (Raw coverage = .20; Consistency = .91) (Table 6).

## Discussion

This study has found high levels of emotional distress in end-of-life patients with COPD and their main family caregivers, who also present high caregiver burden levels. This finding goes in line with previous research carried out with patients at the end of life with different diagnoses and their families [2, 4], but it is new in that it allows the end-of-life context of COPD to be explicitly studied. Regarding some medical variables of the patients, such as time since diagnosis, number of hospital admissions or comorbidity, our data show that the great majority of patients with COPD spend years with this diagnosis, and have been admitted in the hospital several times in advance stage of illness the previous year of the moment of assessment. This data reflects the pattern of relapses characteristic of a patient in advanced COPD, in which there are several exacerbations of the disease that generate intense moments of uncertainty and discomfort for both the patient and their family [6, 13, 14]. The high number of admissions in the final year of life could explain many of the difficulties presented by family caregivers in managing care tasks and making them compatible with their work activity, in addition to the fact that both the patient and the family member face a possible end-of-life situation several times before the patient's death actually occurs.

Regarding the comorbidity and functional independence of the patients, most of the secondary pathologies found in the sample were also common to be found in the normal population with the same age range of the patients, such as diabetes mellitus type II or hypertension [5, 27]. This finding is partly to be expected: since an inclusion criterion was that the patient's main diagnosis should be COPD and no other, those patients with more severe pathologies considered by the medical staff as main pathologies (such as cancer) were not included in the study. At the same time, although the level of functional independence of the patients is variable, only a tiny percentage of them were functionally independent at the time of the assessment, which also tells us about one of the elements related to the burden of the family caregivers: the functional dependence of this patients.

No differences were found in the study variables considering the kinship of the family caregivers. This result may be due, perhaps, to the fact that the vast majority of family caregivers were the daughters or wives of the patients. Therefore, a more diverse sample in this regard would facilitate the detection of possible kinship-related differences. At the same time, the fact that the majority of family caregivers are the wives or daughters of the patients continues to reflect a social reality that, at least in Spain, is very evident: the work of informal caregiving continues to fall under this sociodemographic profile, with the challenges that this implies for women in maintaining their independence and their physical and emotional health [14, 16, 17].

### Emotional distress of end-of-life patients with COPD

In our results, several pathways predict the variance observed in the emotional distress of end-of-life patients with COPD. Based on the linear regression analysis, both the family caregiver's emotional distress and his/her burden predict the distress of the patient, in conjunction with the patient's functional independence and the time living with the disease. The interrelation between the family caregiver's emotional distress and the emotional distress of the end-of-life patient as a dyad goes in line with previous literature [22, 23]. In this study, this interrelation can be observed via the regression analysis as well as with the QCA analysis, and it highlights once more the importance of attending to the emotional needs of patients and their families in the palliative settings, considering the patient and their main family caregiver as a system. Furthermore, the functional independence, comorbidity and time since diagnosis also appear to be closely related to their emotional distress, which is novel in that the comorbid conditions with COPD in this sample do not seem to be particularly severe and yet they contribute greatly to the discomfort of the patient, who is in most cases progressively dependent over several years and suffer several hospital admissions until the time of death. This pattern of several acute phases of the disease, its unpredictability, and its generally long trajectory is one of the things that characterize COPD and define its particular challenges for all involved, including a strong emotional impact on the family system.

### Emotional distress of family caregivers of end-of-life patients with COPD

Several pathways predict the variance observed in the emotional distress of family caregivers of end-of-life patients with COPD. Based on the linear regression analysis and the QCA, the medical variables of the patient, such as time since diagnosis, number of hospital admissions, comorbidity and functional dependence play an essential role in the emotional distress of the family, as does the emotional distress of the patient [22, 23]. The relevance of family caregiver burden is fundamental in preventing their emotional distress, so future studies focused on designing intervention programs in this regard should consider the importance of including elements to reduce the caregiver burden [13, 17, 19, 22].

Therefore, central elements in emotional distress in palliative COPD patients and their families are the caregiver's burden and the patient's functional independence, taking into account that time since diagnosis, number of admissions, and comorbidity also play a role.

### Limitations of the study

One of our study's limitations is that the analysis of possible differences related to the gender of the participants could not be carried out since the vast majority of patients were men, and almost all the family caregivers were women. Future research with greater variability in this regard could further explore possible differences in the study variables that may be related to gender. Another possible limitation of this study is the relatively small sample size. However, few studies have focused on the patient-family caregiver dyad in COPD, while the method and type of analysis used were appropriate for studies with small sample sizes, which mitigates this limitation and gives greater power to our results. Finally, the data from our study is cross-sectional. Therefore, we cannot talk about cause-effect relationships between the variables, just about associations or percentage of observed variance explained by a variable. Future similar research with longitudinal data could shed more light on this regard.

## Conclusions

Our study shows not only that emotional distress in end-of-life situations is an important issue for consideration, but also that the emotional distress of patients and their family caregivers must be considered together, as the respective levels of such distress are closely linked. Particularly crucial for reducing emotional distress in this day and age is the role of caregiver burden, so future lines of research aimed at reducing the emotional distress of the palliative COPD patient and their families must take into account this important element of intervention.

Opportunities for future research include extending the application of our findings with patients with advanced COPD at the end of life and their family caregivers, by comparing them with similar cases related to other pathologies. All of this could contribute to achieving the aims of providing better care to families in end-of-life situations and of reducing their distress more precisely and efficiently.

## Supporting information

S1 Dataset Data_COPD. (DAT)
